# Radiological Changes in the Spinal Cord and Brain of Patients with HTLV-1-Associated Myelopathy/Tropical Spastic Paraparesis (HAM/TSP)

**DOI:** 10.3390/pathogens13110920

**Published:** 2024-10-22

**Authors:** Emily H. Stack, Serhat V. Okar, Tianxia Wu, Mallory Stack, Yair Mina, María Gaitán, Shila Azodi, Will Frazier, Joan Ohayon, Irene C. M. Cortese, Daniel S. Reich, Govind Nair, Steven Jacobson

**Affiliations:** 1 Viral Immunology Section, NINDS, National Institutes of Health, 10 Center Drive, Bethesda, MD 20892, USA; emily.stack@nih.gov (E.H.S.); shila.azodi@nih.gov (S.A.); william.frazier@nih.gov (W.F.); 2 Translational Neuroradiology Section, NINDS, National Institutes of Health, 10 Center Drive, Bethesda, MD 20892, USAmaria.gaitan@nih.gov (M.G.); reichds@ninds.nih.gov (D.S.R.); 3 Clinical Trials Unit, NINDS, National Institutes of Health, 10 Center Drive, Bethesda, MD 20892, USA; wuti@mail.nih.gov; 4 Tel Aviv Sourasky Medical Center, Weizmann St 6, Tel Aviv-Yafo 6423906, Israel; 5 Neuroimmunology Clinic, NINDS, National Institutes of Health, 10 Center Drive, Bethesda, MD 20892, USA; ohayonj@ninds.nih.gov (J.O.); corteseir@ninds.nih.gov (I.C.M.C.); 6 qMRI Core, NINDS, National Institutes of Health, 10 Center Drive, Bethesda, MD 20892, USA; bhagavatheeshg@mail.nih.gov

**Keywords:** central nervous system atrophy, spinal cord atrophy, white matter hyperintensities, magnetic resonance imaging, HTLV-1-associated myelopathy

## Abstract

HTLV-1-associated myelopathy/tropical spastic paraparesis (HAM/TSP) is a chronic, progressive neurological disorder and shares many radiological and clinical features with other more prevalent myelopathies. Here, we quantified spinal cord and brain volumes in adults with HAM/TSP in comparison with healthy volunteers (HVs) and individuals diagnosed with relapsing–remitting or progressive multiple sclerosis (RRMS or P-MS). Clinical disability and MRI were assessed in 24 HVs, 43 HAM/TSP subjects, and 46 MS subjects. Spinal cord cross-sectional area (SCCSA) and brain tissue volumes were measured and compared. HAM/TSP subjects had significantly lower SCCSA corresponding to cervical levels 2 and 3 (C2–3) (54.0 ± 8 mm^2^), cervical levels 4 and 5 (C4–5) (57.8 ± 8 mm^2^), and thoracic levels 4 to 9 (T4–9) (22.7 ± 4 mm^2^) and significantly elevated brain white matter hyperintensity (WMH) fraction (0.004 ± 0.008) compared to the HVs (C2–3: 69.4 ± 8 mm^2^, C4–5: 75.1 ± 9 mm^2^, T4–9: 34.1 ± 4 mm^2^; all *p* < 0.0001; and WMH: 0.0005 ± 0.0007; *p* < 0.001). In the HAM/TSP subjects, SCCSA at all levels but not WMH showed a significant correlation with clinical disability scores. WMH in HAM/TSP subjects, therefore, may not be related to clinical disability. SCCSA in our limited RRMS cohort was higher than the HAM/TSP cohort (C2–3: 67.6 ± 8 mm^2^, C4–5: 72.7 ± 9 mm^2^, T4–9: 33.4 ± 5 mm^2^; all *p* < 0.0001) and WMH was lower than in P-MS subjects (*p* = 0.0067). Principal component analysis suggested that SCCSA and WMH may be used to differentiate HAM/TSP from MS. Understanding these differences msay help establish early diagnostic criteria for HAM/TSP patients.

## 1. Introduction

Human T-cell lymphotropic virus type 1 (HTLV-1)-associated myelopathy/tropical spastic paraparesis (HAM/TSP) is a chronic, progressive myelopathy associated with HTLV-1 infection [1]. It is estimated that over 10 million people are infected with HTLV-1 around the world, where in 2–10% of these individuals, it manifests in clinical disease, such as adult-onset T-cell leukemia and HAM/TSP [2,3,4]. HTLV-1 can be spread through the exchange of bodily fluids such as during sexual intercourse, from mother to child, or through blood transfusions from an infected donor [4,5]. HAM/TSP is endemic in several parts of the world, such as the Middle East, South America, and Japan, and in the Indigenous population in the USA and Australia [2,6].

Central nervous system (CNS) damage in HAM/TSP is believed to be mediated by the recruitment, activation, and expansion of HTLV-1-infected CD4+ T-cells and HTLV-1-specific CD8+ cytotoxic T lymphocytes (CTLs) in the intrathecal compartment [7,8,9]. These infiltrates release proinflammatory cytokines, including interferon-gamma (IFN-γ) and tumor necrosis factor-alpha (TNF-α), and promote the secretion of chemokines from resident cells [10]. This creates a self-perpetuating focus of inflammation within the CNS compartment that culminates in bystander damage of neuronal tissue [4,11,12]. Inflammatory changes have been reported in both the spinal cord and brain of individuals with HAM/TSP. By histopathology, Aye et al. (2000) [13] reported that patients with active chronic inflammation in the spinal cord also had perivascular inflammatory infiltrates in the brain.

Radiologically, spinal cord atrophy and brain lesion formation have long been understood to be a feature of HAM/TSP [14,15,16,17,18,19]. Some in vivo studies have reported that the frequency of cerebral white matter hyperintensities (WMHs) is greater in HAM/TSP patients than in healthy controls, but others have reported no difference between patient and control groups [17,20,21]. To date, therefore, no consensus has been reached regarding the prevalence of brain abnormalities in HAM/TSP nor their relation to spinal cord degradation or clinical disability [20,21,22,23]. We have previously demonstrated that spinal cord atrophy can be estimated in vivo with a semi-automated tool that measures spinal cord cross-sectional area (SCCSA) in the cervical and thoracolumbar spine from magnetic resonance imaging (MRI) [15,16,18,24]. Participants diagnosed with HAM/TSP had significant spinal cord atrophy that began in the thoracic region and progressed to the cervical cord. The severity of thoracic cord atrophy was positively correlated with HTLV-1 proviral load, CD8+ T-cell frequency in the cerebrospinal fluid (CSF), and clinical disability scores [15]. By histopathology, spinal cord thinning in HAM/TSP is associated with demyelination and loss of axons, which predominate in the lateral columns [4].

Sometimes, multiple sclerosis (MS), a neuroinflammatory disease affecting the brain and spinal cord, can present as a progressive myelopathy with similar clinical features to HAM/TSP. MS affects more than 2.8 million people worldwide with unclear etiology [25], and studying HAM/TSP in concert with MS can help characterize shared immunopathogenic mechanisms and identify therapies that can be translated from one disease to the other. MRI of the brain and cervical spinal cord is useful for both diagnosing and monitoring disease progression and treatment response [26,27]. Recent studies have also demonstrated that primary and secondary progressive forms of multiple sclerosis (primary progressive MS or PPMS and secondary progressive MS or SPMS, together called progressive MS or P-MS), but not relapsing–remitting MS (RRMS), are associated with c-spine atrophy, particularly at the cervical vertebral body level 4 and 5 corresponding to cervical enlargement [15,24]. In addition to spinal cord atrophy, the MS brain is mainly characterized by WMH, called lesions, as well as significant atrophy of both normal-appearing gray and white matter [28,29]. Since patients with HAM/TSP are often initially misdiagnosed as having P-MS, especially in areas of the world in which HAM/TSP is not endemic, developing tools that may provide clues to differentiate it from other inflammatory myelopathies is important for patient management.

To evaluate the prevalence and role of WMH and atrophy in the CNS of individuals with HAM/TSP in comparison with control subjects with no neurological symptoms (HVs), we retrospectively measured cervical and thoracolumbar SCCSA and brain tissue volumes using routinely acquired MR images. SCCSA was averaged for the regions corresponding to cervical vertebral body levels 2 and 3 (C2–3), cervical levels 4 and 5 (C4–5), and thoracic levels 4 to 9 (T4–9), and brain tissue was segmented into gray matter (GM), white matter (WM), CSF, and WMH. Spinal cord and brain tissue volumes were then correlated to various measures of clinical disability. Group averages for HAM/TSP were also compared to participants clinically diagnosed with MS, and principal component analysis was used to evaluate which radiological variables were relevant to the differentiation of these neurological conditions.

## 2. Materials and Methods

### 2.1. Study Design and Participants

This retrospective study involved individuals with HAM/TSP, RRMS, and progressive MS (P-MS) and HVs (without any neurological symptoms and negative for HTLV and HIV in the blood). Natural history studies in individuals with HAM/TSP and MS were approved by the institutional review board at the NIH (NCT numbers: NCT00034723 and NCT00001248), and participants were included after informed consent was obtained. The diagnosis was confirmed by clinicians using McDonald’s criteria for MS and proviral loads in individuals with HAM/TSP.

### 2.2. Clinical Testing

All participants underwent neurological examinations, and clinical disability was assessed using the expanded disability status scale (EDSS), Scripps neurologic rating scale (SNRS), time to complete a 25-foot walk (T25FW), and time to complete the 9-hole peg test (9-HPT). The HAM/TSP participants were also rated using the Instituto de Pesquisa Evandro Chagas (IPEC) score. Participants with MS were categorized into RRMS, SPMS, and PPMS phenotypes, and participants with SPMS and PPMS were considered together as one group termed progressive MS (P-MS).

### 2.3. MR Imaging and Analysis

All participants underwent 3T MRI of the brain and spinal cord (Siemens Healthcare GmbH, Erlangen, Germany) with a 20-channel or 32-channel head coil and a 24-channel spine matrix coil. To measure spinal cord thinning, T1-weighted scans of the cervical and thoracic spinal cord (3D gradient-recalled echo sequence, repetition time = 8 ms, echo time = 3 ms, flip angle = 18 degrees, 1 mm isotropic resolution, total acquisition time of about 3 min, 30 s each for the C- and T-spine) were obtained for each subject. Cervical and thoracic images were stitched together using table position information on the DICOM headers and scripts written in-house, and spinal cord cross-sectional area (SCCSA) was measured as previously described [15,18,24]. Briefly, the user manually selected the region of the spinal cord corresponding to vertebral body levels C1 and T10 using scripts written in Matlab (MathWorks Inc., Natick, MA, USA). Axial images perpendicular to the selected cord edge were then automatically reformatted at each point and the cross-sectional area was calculated. The analysis results were checked using both manual and automated quality assurance steps, and SCCSA was plotted against the normalized distance from C1 to T10. For statistical comparisons, the SCCSA for each subject was also averaged over the regions corresponding to vertebral levels C2–3, C4–5, and T4–9. These three regions were chosen as they are not affected by inter-subject anatomical differences and have unique pathological and clinical implications; C2–3 comprises predominantly white matter tracts, C4–5 corresponds to cervical enlargement and has higher gray matter content than the C2–3 region, and T4–9 houses white matter tracts for lower extremity innervation and has been shown to be the first region to degrade in HAM/TSP [15]. Due to it either not being possible to complete a scan or due to poor scan quality, SCCSA data were not acquired for 8 healthy controls, 3 HAM/TSP subjects, 4 RRMS subjects, and 3 P-MS subjects.

To evaluate brain tissue volumes, the machine learning program Classification Using DErivative Based Features (C-DEF) [30,31] was used to perform automated brain segmentations from the MR images. T1-weighted (T1 magnetization-prepared rapid gradient echo with 2 inversion preparations or MP2RAGE, inversion-prepared turbo-flash or IR-TFL sequence, repetition time (TR)/echo time (TE)/inversion times (TI1 and TI2) = 5000/2.9/700/2500 ms, FA = 4.5 deg, 176 slices, 1 mm isotropic resolution, acquisition time = 8 min 20 s) and fluid-attenuated inversion recovery (FLAIR, 3D IR-TSE sequence, TR/TE/TI = 4800/352/1800 ms, 176 slices, 1 mm isotropic resolution, acquisition time = 5 min 22 s) contrasts were obtained for each subject and analyzed using the C-DEF algorithm as previously described [30]. In brief, models were trained on participants with variable lesion severity; two separate models were created for scans acquired with 20-channel and 32-channel head coils. An analysis was conducted using voxel data from the MP2RAGE sequence (including the two inversion images and the denoised T1-weighted image) and FLAIR images, and brain tissue was segmented into GM, WM, CSF, and white matter hyperintensities (lesions). All segmentations were quality-checked by S.V.O., a trained neurologist with 6 years of experience in MS imaging. Segmentations that failed quality assurance (13/113 scans), which was typically due to the misidentification of large lesions as GM, were manually corrected using 3DSlicer. Manual segmentations were performed by E.H.S. and quality checked by S.V.O. All tissue volumes were normalized to intracranial volume to give brain fractions and adjusted for age. All brain scans analyzed were acquired within one day of the subject’s spinal scans.

### 2.4. Statistical Methods

Box–Cox transformation was applied to the outcome variables with non-normal distribution: the natural logarithm for lesion fraction and pro-viral load in the peripheral blood mononuclear cells (PVL-PBMCs), inverse transformation for T25FW, and square transformation for average 9-HPT. The Shapiro–Wilk test was used to test the normality of the (Studentized) residuals. For each of the 11 outcome variables, analysis of covariance (ANCOVA) was applied to examine the group-wise differences using Tukey’s method to adjust for multiple comparisons. Sex and age were considered as covariate variables, for which variables with *p* > 0.1 were dropped from the model. Pearson simple and partial correlation analyses were conducted to examine the association between the 4 brain variables and the 3 spine variables with age as a covariate (sex had no effect on any outcome variables) for each of the 4 clinical groups separately. These correlation analyses were also applied to examine the association between the 4 clinical variables and the 3 spine variables. Finally, principal component analysis was performed using three spine variables (C4–5, C2–3, and T4–9) and three lesion variables (the log-transformed number of brain lesions, the log-transformed lesion fraction, and the log-transformed median lesion volume of each participant), where the number of lesions and lesion fraction were pre-adjusted for age (the other 4 variables were not associated with age). Varimax rotation was applied to derive orthogonal (uncorrelated) components, which were used to evaluate the group-wise difference based on ANOVA and Tukey’s method. SAS version 9.4 was used for all analyses, and a *p*-value < 0.05 was considered to be statistically significant.

## 3. Results

### 3.1. Participants

In total, 113 individuals were included in the study, comprising 24 HVs, 43 individuals with HAM/TSP, 26 individuals with RRMS, and 20 individuals with P-MS. Demographic and clinical information for all cohorts are summarized in Table 1.

### 3.2. Spinal Cord Atrophy

**Figure 1:**
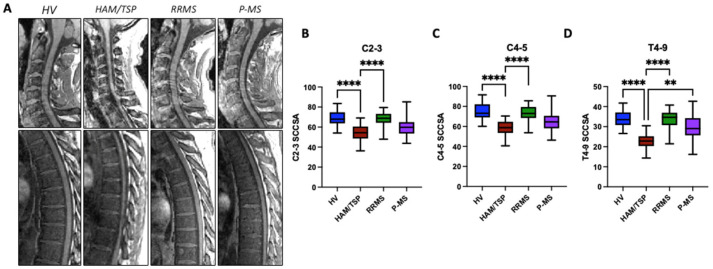
Global spinal cord atrophy. (**A**) Representative mid-sagittal T1-weighted MR images of the cervical (upper row) and thoracolumbar (lower low) from healthy volunteers (HVs), HTLV-1-associated myelopathy/tropical spastic paraparesis (HAM/TSP), relapsing–remitting multiple sclerosis (RRMS), and progressive multiple sclerosis (P-MS). Group-averaged spinal cord cross-sectional areas (SCCSA) from (**B**) C2–3, (**C**) C4–5, and (**D**) T4–9 showing significantly lower values in the HAM/TSP subjects at all regions of the cord. ** *p* < 0.01; **** *p* < 0.0001.

As shown in the representative mid-sagittal MR images of the cervical and thoracolumbar spine for one subject from each group, there is visible spinal cord thinning in the HAM/TSP subjects (Figure 1A). To quantitatively compare the degree of spinal cord thinning across the groups, the average SCCSA was calculated for three representative regions of the cord corresponding to vertebral body levels C2–3, C4–5, and T4–9 (Figure 1B–D). HAM/TSP subjects had significantly lower average SCCSA at C2–3 (54.0 ± 8 mm^2^), C4–5 (57.8 ± 8 mm^2^), and T4–9 (22.7 ± 4 mm^2^) compared to the HVs (C2–3: 69.4 ± 8 mm^2^, C4–5: 75.1 ± 9 mm^2^, T4–9: 34.1 ± 4 mm^2^; *p* < 0.0001) and RRMS (67.6 ± 8 mm^2^, 72.7 ± 9 mm^2^, 33.4 ± 5 mm^2^; *p* < 0.0001) averages (Figure 1B–D). There were no statistically significant differences in average SCCSA for the RRMS or P-MS subjects (C2–3: 60.7 ± 11 mm^2^; C4–5: 66 ± 13 mm^2^, T4–9: 29.7 ± 7 mm^2^; *p* > 0.05) compared to the HVs, but a trend toward lower area at C2–3 was observed in the P-MS subjects (*p* = 0.062). There were no significant correlations between SCCSA at any region and age or sex.

### 3.3. Brain Lesion Volume and Atrophy

**Figure 2:**
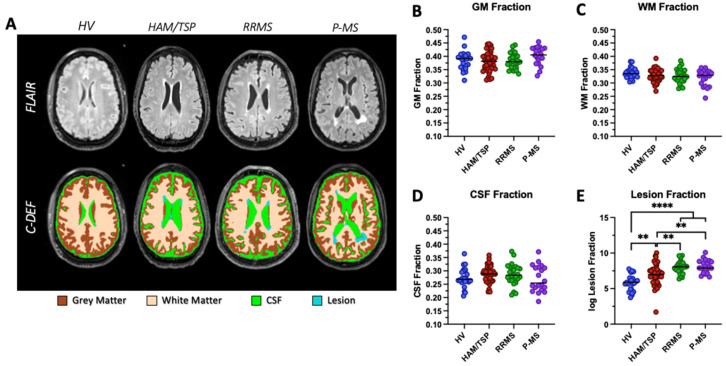
Brain volumes. (**A**) FLAIR image (top row) and brain segmentation results overlaid on FLAIR images showing segmented (bottom row) grey matter (brown), white matter (beige), cerebrospinal fluid (CSF, green), and white matter hyperintensities or lesions (teal) from a representative participant in each diagnosis group. Group-averaged analysis of (**B**) grey matter, (**C**) white matter, (**D**) CSF, and (**E**) lesion or white matter hyperintensity volumes expressed as a fraction of total intracranial volume and adjusted for age showing differences only in the WMH volumes. ** *p* < 0.01; **** *p* < 0.0001.

Brain segmentations were completed on all 113 individuals. FLAIR images and C-DEF segmentation masks from a representative participant in each diagnosis group are shown in Figure 2A. Tissue was segmented into GM (brown), WM (beige), CSF (green), and lesions (blue) and normalized to total intracranial volume to obtain brain fractions. All brain tissue fractions were significantly correlated with age (GM fraction, *p* < 0.0001; WM fraction, *p* = 0.0008; CSF fraction, *p* < 0.0001; lesion fraction, *p* < 0.0001); therefore, age-adjusted brain fractions were used for downstream statistical analyses. No statistically significant differences in age-adjusted GM fraction, WM fraction, or CSF fraction were observed between the HAM/TSP subjects and the other groups in our cohort (Figure 2B–D). A significant increase in average age-adjusted lesion fraction was observed in HAM/TSP (0.004 ± 0.008), RRMS (0.004 ± 0.004), and P-MS (0.0087 ± 0.014) subjects compared to the HVs (0.0005 ± 0.0007; all *p* < 0.002, Figure 2E). The average age-adjusted lesion fraction in HAM/TSP subjects was significantly lower than both RRMS and P-MS averages (*p* = 0.0067, *p* = 0.0014, respectively, Figure 2E).

### 3.4. Correlation between Radiological and Clinical Measures

On average, participants with HAM/TSP and P-MS had more severe clinical disability than participants with RRMS as indicated by significant increases in median EDSS (6.5, 6, and 1.75, respectively; *p* < 0.0001) and significant decreases in median SNRS (69, 65, and 93, respectively; *p* < 0.0001, Table 1). Pearson partial correlation coefficients for the relationship between SCCSA or brain lesion fraction and all disability measures are summarized in Table 2.

**Figure 3:**
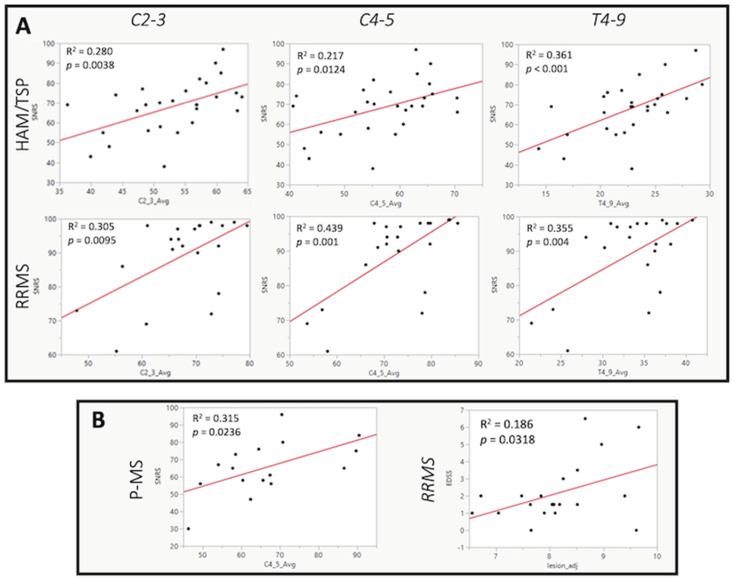
Correlation of radiological and clinical scores. (**A**) Plots showing a statistically significant correlation (partial Pearson’s) between Scripps neurologic rating scale (SNRS) and spinal cord cross-sectional area (SCCSA) at all cord levels (in columns) for HAM/TSP (top row) and RRMS (bottom row) subjects with disability increasing with reducing SCCSA. (**B**) SCCSA showed a significant correlation only in the C4–5 region in the P-MS group (left). Brain white matter hyperintensity (or lesion) volumes were significantly correlated with a clinical disability only in the relapsing–remitting multiple sclerosis group (correlation with EDSS shown on the right).

In both HAM/TSP and RRMS subjects, SCCSA at all regions of the cord was significantly positively correlated with SNRS score (Table 2, Figure 3A), indicating that participants with thinner spinal cords had worsened clinical disability. In HAM/TSP subjects, the strongest correlation was observed at the level of T4–9 (r = 0.60, *p* < 0.001, Table 2, Figure 3A). This region was also significantly negatively correlated with EDSS (r = −0.40, *p* = 0.0331) and T25FW (r = −0.55, *p* = 0.0031). The cervical enlargement was the only level of the cord to have a significant correlation between cross-sectional area and SNRS in P-MS subjects (r = 0.56, *p* = 0.0221, Figure 3B). Importantly, brain lesion fraction was not correlated with any disability rating scale in HAM/TSP subjects (Table 2). In contrast, in RRMS subjects, higher brain lesion fraction was significantly correlated with increasing EDSS (r = 0.47, *p* = 0.0318), T25FW (r = 0.56, *p* = 0.0031), and 9-HPT (r = 0.48, *p* = 0.0234, Table 2 and Figure 3B). SCCSA at C2–3 and T4–9 was also significantly correlated with IPEC score. However, neither SCCSA at any level nor brain lesion volume showed any correlation with PVL-PBMC or disease duration. The correlation between PVL in the peripheral blood and SCCSA at C2–3 and T4–9 levels approached significance (*p* = 0.08 for both).

### 3.5. Principal Component Analysis Results

**Figure 4:**
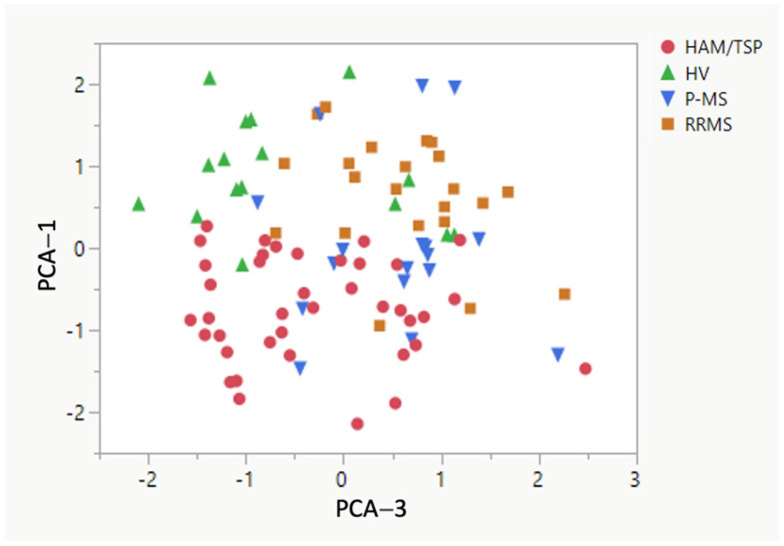
Principal component analysis. Principal components 1 (dominated by SCCSA) and 3 (dominated by median WMH volume from individuals) derived from brain and spine radiological variables show the ability to separate the diagnosis groups.

As shown in Figure 4, principal component analysis demonstrated that the radiological variables were clustered into three principal components. The first component contained all three regional SCCSA measures while the second and third components contained information pertaining to brain lesion load (lesion fraction and number of radiological hyperintensities in component two; median volume of radiological hyperintensities in component three). Component one, SCCSA measures, was sufficient to differentiate the HAM/TSP group from all other groups (HVs, *p* < 0.0001; RRMS, *p* < 0.0001; and P-MS, *p* = 0.0031, Figure 4). Component three, volume of radiological hyperintensities in the brain, was also able to separate the HAM/TSP group from the RRMS and P-MS groups (*p* < 0.001 and *p* < 0.01, respectively) but not from the HVs (*p* = 0.633).

## 4. Discussion

The radiological hallmark of HAM/TSP is spinal cord thinning, but conflicting evidence has been reported regarding the prevalence of brain abnormalities in the disease. Using routinely acquired MR images, we retrospectively measured spinal cord thinning and brain lesion volume and atrophy in HAM/TSP subjects compared to control participants to evaluate the degree of damage to each compartment and its correlation with clinical disability measures. In addition, these metrics were also compared to a limited cohort of participants clinically diagnosed with MS to look for patterns of radiological changes in various progressive myelopathies.

We have previously shown that subjects with HAM/TSP have thinner spinal cords than healthy subjects and subjects with RRMS [15,16,18,24]. More severe thinning in the thoracic cord was correlated with both worsened clinical disability and elevated immune markers [15]. In agreement with our previous reports, herein, we demonstrate significantly lower average SCCSA at the vertebral body levels of C2–3, C4–5, and T4–9 in subjects with HAM/TSP compared to healthy volunteers and subjects with RRMS (Figure 1B–D).

Based on our previous spinal analysis, it was of interest to evaluate if there were radiological abnormalities in the brain of HAM/TSP subjects. We used C-DEF, a machine-learning program that has been previously validated in MS and people living with human immunodeficiency virus (HIV), to perform automated brain segmentations and tissue volume measurements from MR images [30,31,32]. Brain tissue was segmented into GM, WM, CSF, and lesions, normalized to total intracranial volume, and adjusted for age. Significantly, we found that subjects with HAM/TSP had an increase in age-adjusted brain lesion fraction compared to healthy controls, though it was lower than both RRMS and P-MS averages (Figure 2E). There was no evidence of GM, WM, or whole-brain atrophy in HAM/TSP subjects compared to the control subjects. This finding is consistent with previous radiological and histopathological studies that have reported the occurrence of brain lesions, but not atrophy, in a subset of subjects with HAM/TSP [21,22,23,33]. Kalil et al. (2021) [21] evaluated the occurrence of brain white matter hyperintensities using MRI in 22 subjects with HAM/TSP compared to healthy controls and asymptomatic carriers of HTLV-1. They reported that lesions were more frequent in HAM/TSP subjects than in asymptomatic carriers and occurred preferentially in periventricular white matter. Another study also reported a higher frequency of white matter lesions using MRI in a cohort of 28 HAM/TSP subjects, but the observation that lesions predominated in older subjects suggested that they may reflect age-related degenerative processes rather than disease-specific pathology [23]. These initial studies, however, relied on low sample sizes and a binary assessment of lesions as present or absent. We extended these early findings by evaluating a larger HAM/TSP cohort, measuring lesion volume using a novel machine learning algorithm to reflect lesion burden more completely, and adjusting all brain measures for age to control for age-related degenerative processes. After controlling for these confounding variables, we still found a significant increase in lesion burden in our HAM/TSP cohort compared to the control subjects.

Evidence of inflammatory changes in the brain of HAM/TSP subjects has also been found in histopathological analyses [13,22]. Aye et al. (2000) [13] reported that HAM/TSP subjects with active chronic spinal cord lesions had perivascular inflammatory infiltrates in the brain, but those with inactive chronic spinal cord lesions did not. Brain parenchymal infiltration was minimal and primarily comprised CD8+ T-cells, which also predominated in the spinal cord. Other studies reported that brain lesions on MRI were associated with demyelination, astrocytic gliosis, and hyaline thickening of small vessels but not inflammatory cell infiltration [22]. These studies suggest that inflammation is likely not restricted to the spinal cord in HAM/TSP, but that inflammatory changes in the brain may not be as severe or diffuse as those in the cord.

To explore the relationship between damage to each anatomical region and clinical disability, we evaluated the correlation between spinal cord thinning or brain lesion fraction and score on various clinical disability scales. All subjects were rated using the EDSS, SNRS, T25FW, and the 9-HPT, and HAM/TSP subjects were also evaluated using the IPEC score. In HAM/TSP subjects, we observed a significant correlation between spinal cord thinning in every region and worsened clinical disability (Table 2). For most clinical variables examined, the strongest correlation was observed in the thoracic cord. This finding is consistent with the previous literature, which reports that the thoracic cord is the first to degrade in HAM/TSP and is the region with the most prominent immune cell infiltration, inflammation, demyelination, and thinning [13,14,15]. The relationship between PVL-PBMC and SCCSA at C2–3 and T4–9 approached significance at a *p*-value of 0.08. Indeed, it needs to be pointed out that there was no significant correlation seen between SCCSA and disease duration, which ranged from 1.2 years to 34.1 years (median of 7.4 years) in this cohort. Indeed, the clinical progression rate is known to be different in different patients and ranges from rapid progressors to patients who show no progression for decades. Moreover, and perhaps more importantly, the start of symptoms for patients can be a very subjective measure, with some patients being acutely aware of symptoms and others not.

Importantly, there were no statistically significant correlations between age-adjusted brain lesion fraction and any clinical disability measure in HAM/TSP subjects. Collectively, these results suggest that although HAM/TSP subjects may develop brain lesions at a greater rate than healthy ageing populations, brain lesions are less likely to be related to clinical disability in HAM/TSP.

Finally, we compared the number of radiological hyperintensities and their average volume in each of our groups. Using principal component analysis, we demonstrated that spinal cord thinning was sufficient to differentiate subjects with HAM/TSP from all other groups. Interestingly, we also found that the volume of radiological hyperintensities separated HAM/TSP subjects from subjects with both RRMS and P-MS. This suggests that in the absence of complete spinal cord imaging and volume measures, the evaluation of brain white matter hyperintensities may aid in the differentiation of HAM/TSP from progressive forms of MS. This is clinically significant as MS, particularly the primary progressive phenotype, is included in the differential diagnosis of HAM/TSP. In our experience, many of our HAM/TSP subjects have once carried a diagnosis of progressive MS. Current methods to differentiate these myelopathies include serological and polymerase chain reaction (PCR) testing of the blood and CSF for the presence of HTLV-1, which is not routinely performed in a clinical setting. Expanding our repertoire of tools that can help differentiate these conditions may, therefore, improve the speed and accuracy of diagnosis. Since there are no known treatments that promote remyelination or CNS repair, early and accurate diagnosis is critical to ensure that individuals with chronic progressive myelopathies begin disease-modifying therapies as early as possible in the disease course to help prevent irreversible neurological damage.

The present study has several limitations. The major limitation of this study is the small sample size in the MS groups. The small sample size may result in increased variability, especially when adjusting for age. Indeed, the age of participants in the HV, HAM, and P-MS groups was different and could have contributed to some of the negative results seen in the MS groups. Future studies will focus on including more subjects in each group that have brain and spinal cord scans performed concurrently. Secondly, it is well known that the clinical rating scales included herein are skewed toward rating motor symptoms [34]. We did not include any rating of cognitive function; therefore, we cannot comment on any relationship that may exist between the radiological variables measured herein and cognitive impairment, which has been reported in a subset of HAM/TSP subjects [21,35]. As asymptomatic carriers of HTLV-1 are rarely seen in our clinics, we were unable to evaluate if the elevated brain lesion fraction reported herein is related to the pathogenesis of HAM/TSP or more broadly to infection with HTLV-1.

## 5. Conclusions

We have shown that the cohort of HAM/TSP subjects had both spinal cord atrophy and elevated age-adjusted brain lesion fraction compared to healthy volunteers. The average brain lesion fraction in HAM/TSP subjects was significantly lower than both RRMS and progressive MS averages. In HAM/TSP subjects, we found strong correlations between spinal cord thinning at all regions and worsened clinical disability, with the strongest correlations being observed in the thoracic cord. Importantly, we did not observe any correlation between brain lesion fraction and any measure of clinical disability in HAM/TSP subjects. Using principal component analysis, we demonstrated that spinal cord thinning can differentiate the HAM/TSP group from all other groups and the median volume of radiological hyperintensities can differentiate HAM/TSP from both RRMS and P-MS. These findings suggest that individuals with HAM/TSP may develop brain lesions at a greater rate than can be attributed to age-related degenerative processes, but these brain lesions may not be related to clinical disability in this patient population. The present work reaffirms the importance of measuring spinal cord thinning as an indicator of disease progression and severity in HAM/TSP and suggests that in the absence of complete cervical and thoracolumbar spine data, radiological hyperintensities in the brain may be a useful disease marker.

## Figures and Tables

**Table 1:** Demographic and clinical information.

Diagnosis	N (% Female)	Age (Years) Mean ± SD	EDSS Med (IQR)	SNRS Med (IQR)	IPEC Med (IQR)	T25FW (s) Med (IQR)	9-HPT (s) Med (IQR)
HAM/TPS	43 (77)	58 ± 12	6.5 (0.4)	69 (17)	15.5 (6.5)	16.5 (14.8)	21.7 (5.1)
RRMS	26 (69)	54 ± 12	1.75 (0.5)	93 (13)	--	4.9 (1.1)	19.0 (4.4)
P-MS	20 (70)	61 ± 8	6 (1.25)	65 (18)	--	10.9 (10.4)	26.9 (15.4)
SPMS	13 (69)	60 ± 8	6 (0.5)	65 (15)	--	11.1 (11.5)	29.1 (16.3)
PPMS	7 (71)	63 ± 9	5 (2.5)	71 (24)	--	10.7 (5.1)	23.5 (6.7)
HVs	24 (63)	51 ± 13	--	--	--	4.6 (0.7)	18.5 (2.4)

Diagnosis groups: HTLV-1-associated myelopathy/tropical spastic paraparesis (HAM/TSP), relapsing–remitting multiple sclerosis (RRMS), progressive multiple sclerosis (P-MS) consisting of secondary progressive MS (SPMS) and primary progressive MS (PPMS), and healthy volunteers (HVs). Clinical scales: Expanded disability status scale (EDSS), Scripps neurologic rating scale (SNRS), Instituto de Pesquisa Evandro Chagas (IPEC) score, time to complete a 25-foot walk (T25FW), and time to complete the 9-hole peg test (9-HPT).

**Table 2:** Partial correlation coefficients between radiological and clinical measures.

Group	Region	EDSS	SNRS	IPEC	T25FW	9-HPT
HAM/TSP	C2–3	−0.38 *	0.53 **	−0.36 *	−0.37	−0.27
	C4–5	−0.26	0.47 *	−0.22	−0.25	−0.19
	T4–9	−0.40 *	0.60 ***	−0.35 *	−0.55 **	−0.13
	WMH	0.00	0.05	--	0.26	0.09
RRMS	C2–3	−0.16	0.55 **	--	−0.11	0.24
	C4–5	−0.27	0.66 ***	--	−0.19	0.24
	T4–9	−0.25	0.60 **	--	−0.31	0.02
	WMH	0.47 *	−0.32	--	0.56 **	0.48 *
P-MS	C2–3	−0.27	0.34	--	−0.17	−0.31
	C4–5	−0.37	0.56 *	--	−0.28	−0.38
	T4–9	−0.23	0.37	--	−0.12	−0.15
	WMH	−0.42	−0.02	--	−0.22	0.13

Diagnosis groups: HTLV-1-associated myelopathy/tropical spastic paraparesis (HAM/TSP), relapsing–remitting multiple sclerosis (RRMS), and progressive multiple sclerosis (P-MS consisting of secondary progressive and primary progressive MS). Clinical scales: Expanded disability status scale (EDSS), Scripps neurologic rating scale (SNRS), Instituto de Pesquisa Evandro Chagas (IPEC) score, time to complete a 25-foot walk (T25FW), and time to complete the 9-hole peg test (9-HPT). * *p* < 0.05; ** *p* < 0.01; *** *p* < 0.001.

## Data Availability

All data and software used in this study can be shared upon request, according to the data sharing policy of the National Institute of Health and as per the specific study protocol.
